# Glycolysis-Related Gene Expression Profiling Screen for Prognostic Risk Signature of Pancreatic Ductal Adenocarcinoma

**DOI:** 10.3389/fgene.2021.639246

**Published:** 2021-06-23

**Authors:** Wenjing Song, Xin He, Pengju Gong, Yan Yang, Sirui Huang, Yifan Zeng, Lei Wei, Jingwei Zhang

**Affiliations:** ^1^Department of Breast and Thyroid Surgery, Hubei Key Laboratory of Tumor Biological Behaviors, Hubei Cancer Clinical Study Center, Zhongnan Hospital, Wuhan University, Wuhan, China; ^2^Department of Pathology and Pathophysiology, School of Basic Medical Sciences, Wuhan University, Wuhan, China

**Keywords:** glycolysis, gene signature, overall survival, prognostic biomarker, pancreatic ductal adenocarcinoma

## Abstract

**Objective:** Pancreatic ductal adenocarcinoma (PDAC) is highly lethal. Although progress has been made in the treatment of PDAC, its prognosis remains unsatisfactory. This study aimed to develop novel prognostic genes related to glycolysis in PDAC and to apply these genes to new risk stratification.

**Methods:** In this study, based on the Cancer Genome Atlas (TCGA) PAAD cohort, the expression level of glycolysis-related gene at mRNA level in PAAD and its relationship with prognosis were analyzed. Non-negative matrix decomposition (NMF) clustering was used to cluster PDAC patients according to glycolytic genes. Prognostic glycolytic genes, screened by univariate Cox analysis and LASSO regression analysis were established to calculate risk scores. The differentially expressed genes (DEGs) in the high-risk group and the low-risk group were analyzed, and the signal pathway was further enriched to analyze the correlation between glycolysis genes. In addition, based on RNA-seq data, CIBERSORT was used to evaluate the infiltration degree of immune cells in PDAC samples, and ESTIMATE was used to calculate the immune score of the samples.

**Results:** A total of 319 glycolysis-related genes were retrieved, and all PDAC samples were divided into two clusters by NMF cluster analysis. Survival analysis showed that PDAC patients in cluster 1 had shorter survival time and worse prognosis compared with cluster 2 samples (*P* < 0.001). A risk prediction model based on 11 glycolysis genes was constructed, according to which patients were divided into two groups, with significantly poorer prognosis in high-risk group than in low-risk group (*P* < 0.001). Both internal validation and external dataset validation demonstrate good predictive ability of the model (AUC = 0.805, *P* < 0.001; AUC = 0.763, *P* < 0.001). Gene aggregation analysis showed that DEGs highly expressed in high-risk group were mainly concentrated in the glycolysis level, immune status, and tumor cell proliferation, etc. In addition, the samples in high-risk group showed immunosuppressed status and infiltrated by relatively more macrophages and less CD8+T cell.

**Conclusions:** These findings suggested that the gene signature based on glycolysis-related genes had potential diagnostic, therapeutic, and prognostic value for PDAC.

## Introduction

Pancreatic ductal adenocarcinoma (PDAC) is the most common type of pancreatic cancer, accounting for about 90% of pancreatic cancer cases. PDAC is a highly fatal cancer with a 5-year overall survival rate of 6% (2–9%) (Ilic and Ilic, 2016). By 2030, PDAC is projected to be the second leading cause of cancer-related deaths (Rahib et al., 2014). Surgical resection is considered to be the only treatment for PDAC, which can significantly prolong the survival time. However, due to the hidden onset, rapid progression, early metastasis and lack of sensitive screening methods (Singhi et al., 2019), most PDAC are confirmed in its advanced stage and lost the thus become unresectable. Therefore, about 80% of people diagnosed with PDAC will die within a year and thus early diagnosis of PDAC is particularly important (Zhang et al., 2017). For diagnostic methods, the specificity and sensitivity of tumor biomarkers for predicting PDAC are not satisfactory (Zhou et al., 2015), and it is difficult to find small lesions by computed tomography (CT) (Fox et al., 2016; Zhang et al., 2017). Although pathological examination is the gold standard for the diagnosis of PDAC, the deep location of pancreas limits its application. Therefore, it is urgent to develop a method and strategy for early detection of PDAC, which will be beneficial to the diagnosis and treatment of PDAC patients. In addition, radiotherapy, chemotherapy, targeted molecular therapy and immunotherapy have also been shown to be effective in the treatment of PDAC (Kamisawa et al., 2016). The diversity of treatment options requires more individualized management of PDAC patients, which can improve both the treatment of cancer and the quality of life. Tumor progression and drug response in PDAC patients are closely related to the molecular characteristics, phenotypic differences and tumor microenvironment (TME). According to these characteristics, different classification systems for PDAC subtypes could be established to predict the prognosis of patients and select therapeutic drugs and therapies (Collisson et al., 2011; Moffitt et al., 2015; Bailey et al., 2016; Puleo et al., 2018). Advances in tumor molecular biology have led to the development of new predictive tools based on prognostic genes. These prognostic markers reflecting tumor progression at the molecular level may contribute to more accurate personalized survival prediction.

Microenvironment is the cellular environment where cells grow, proliferate and invade, and tumor cells are no exception (Wang et al., 2018). Most solid tumors rely heavily on aerobic glycolysis for energy production due to the metabolic reprogramming of tumor cells to facilitate the aerobic glycolysis process to adapt to their heterogeneous microenvironment. In addition to the tumor cells, the TME also includes the surrounding immune cells, fibroblasts and immune cells. Because of the dense connective tissue and vascular microenvironment of PDAC, PDAC cells are difficult to penetrate and in a low-perfusion environment, which promotes metabolic rearrangement in the PDAC so that tumor cells can make full use of oxygen even in a hypoxia state (Fu et al., 2018; Weniger et al., 2018). Thus, PDAC generally displays enhanced glycolysis, including overexpression of glycolytic enzymes and increased lactic acid production, which is caused by mitochondrial dysfunction, abnormal expression of oncogenic genes, specific transcription factors, hypoxic tumor microenvironment, and tumor-associated macrophage (Cheng et al., 2019; Karasinska et al., 2020; Yang et al., 2020). This energy metabolic pathway not only provides energy to cancer cells, but also produces metabolic intermediates that promote cell proliferation, invasion and drug resistance of cancer (Deberardinis et al., 2008). Reprogramming of metabolism in cancer cells is regulated by multiple factors and signaling pathways, such as hypoxia inducible factor (HIF-1), Myc, p53, and the PI3K/Akt/mTOR pathway (Pelicano et al., 2006; Dang et al., 2009; Liao et al., 2009; Masui et al., 2013). Some preclinical and clinical studies have shown that drugs targeting these factors and signaling pathways and the use of anti-glycolysis agents to deprive the basic metabolic needs of cancer cells and interfere with cancer growth are effective as therapies to inhibit cancer progression (Abdel-Wahab et al., 2019; Jagust et al., 2019). In addition, it is gratifying to note that anti-glycolysis agents have been found to have the potential to increase the sensitivity of cancer cells and improve treatment resistance. Therefore, it is of great significance to search for molecules related to glycolysis and explore their expression characteristics and functional involvement in PDAC.

In the present study, 11 prognostic glycolysis-related genes were selected based on high-throughput sequencing results and clinicopathological features of the PDAC data sets, and the prognostic gene signature was proposed, which was used to describe the glycolytic level in PDAC samples. Multivariate Cox regression confirmed that prognostic gene signature was independent prognostic influential factor of PDAC, and was validated using GEO data sets. In addition, we described the gene expression at the protein level, and investigated in depth the correlation between the signal pathways involved in the gene signature and biochemical processes and tumor immunity. These results may be helpful for the study of glycolysis-related molecules in PDAC.

## Methods

### Patient Data Acquisition

The Cancer genome atlas (TCGA) (https://portal.gdc.cancer.gov/) is a cancer research project established by the national cancer institute and the national human genome research institute jointly, covering 33 kinds of cancer. Download RNA-seq and corresponding clinical data of PDAC patients (survival time, survival status, diagnostic age, gender, history of smoking, history of alcohol, history of diabetes, history of chronic pancreatitis, tumor site, histological grade, pathological T, N, M, stage, residual tumor and radiation therapy). After data cleaning, a total of 173 patients with PDAC had complete survival data, and 73 patients had complete clinicopathological data. Data download and online analysis has been commenced on 1 November 2020 (TCGA: V21.0).

### Identification of Glycolysis-Related Genes

The Molecular Signature Database (MSigDB) (https://www.gsea-msigdb.org/gsea/msigdb/index.jsp) (Subramanian et al., 2005; Liberzon et al., 2011, 2015) provides a collection of annotated gene sets to analyze. Search for “glycolysis” by keyword, download all glycolysis-related data sets, extract and sort out the contained genes. Then, we performed Gene Ontology (GO) and Kyoto Encyclopedia of Genes and Genomes (KEGG) enrichment analyses on these 319 glycolysis-related genes to verify whether these genes are involved in regulating the process of glycolysis.

### PDAC Subclasses Identification Based on Glycolysis-Related Genes

The obtained 319 glycolysis-related genes were used for non-negative matrix factorization (NMF) clustering. The purpose of NMF was to identify potential features in the gene expression profile by decomposing the original matrix into different non-negative matrix. Use the “NMF” R package to perform unsupervised NMF clustering with 1,000 repeat samples and a maximum grouping of 6 on the metadata set. The cumulative distribution function (CDF) and consensus heatmap were used to evaluate the optimal *k* value, and the TCGA-PAAD samples were divided into different clusters according to the glycolysis level of tumor tissues.

### Prognosis Analysis

Kaplan-Meier (K-M) method was used to plot the survival curves between the expression level of 319 glycolysis-related genes and the overall survival time (OS) of PDAC patients (cutoff by the median expression level). The significance of the difference was tested by log-rank test, and the genes whose *P* < 0.01 were screened for subsequent study. Differences in OS, histological grade and pathological stage between the two groups were compared and visualized by box plot using R language (version 3.6.1).

### Gene Signature Identification and Score Construction

The prognostic related genes were identified by univariate Cox regression analysis. After that, least absolute shrinkage and selection operator (LASSO) regression was employed to identify independent prognostic influencing genes powerfully associated (*P* < 0.05) with OS in PDAC patients. The risk score was calculated by the following formula:

$$\begin{array}{l} \text{Risk score}=\sum_{i\;=\;1}^{n}Coef\left(i\;\right)X\left(i\right) \end{array}$$

What needs to be commented is that “*n*” represents the count of genes in the model, “*Coef (i)*,” that is “coefficient,” represents the coefficient of each gene, *X (i)* means the mRNA expression level of each gene. When the coefficient is >0, it indicates that the overexpression of this gene increases the risk of PDAC patients, otherwise, it means that the gene has a protective effect on patients with PDAC. Download the Gene Expression Omnibus (GEO) data set (GSE62452) (Yang et al., 2016) to externally validate the model. Patients in TCGA-PAAD and GEO were divided into low-risk group and high-risk group according to the median risk level, respectively. The survival differences of the two groups were compared and visualized with the survival status plot, risk heatmap and survival curve. The area under the curve (AUC) of the 95% confidence interval was determined according to the receiver operating characteristic curve (ROC), and the accuracy and specificity of the model were quantitatively evaluated. Diagnosis of age, gender, history of smoking, history of alcohol, history of diabetes, history of chronic pancreatitis, tumor site, histological grade, pathological T, N, M, stage, residual tumor, radiation therapy and risk score were all included in this study for univariate and multivariate Cox regression analysis, which determined that risk score based on 11 glycolysis-related genes was the independent prognostic factor for PDAC.

### Functional Inference

Gene Set Enrichment Analysis (GSEA), a desktop software used to analyze gene sets, can be downloaded from the Broad Institute GSEA website (https://www.gsea-msigdb.org/gsea/index.jsp) (Subramanian et al., 2005). To infer functional annotations of 11 glycolysis-related genes, GO enrichment [c5.all.v7.2.symbols.gmt (Gene oncology)] and KEGG pathway analysis [c2.cp.kegg.v7.2.symbols.gmt (Curated)] of differentially expressed genes (DEGs) in low-risk group and high-risk group were enriched by GSEA (version 4.0.1). ChIP - X Enrichment Analysis Version 3 (ChEA3) (https://maayanlab.cloud/chea3/#top) (Keenan et al., 2019) is a web-based enrichment tool of transcription factor (TF) analysis. We predicted the top 25 TF that are most closely related to the 11 glycolysis-related genes by using Fisher's precise test.

### Analysis of Tumor Infiltrate Immune Cells in PDAC

Based on RNA-seq data, the “Cibersort” R package was used to estimate the abundance of 22 TIICs in a single sample (Newman et al., 2019), and the stromal, immune and estimated scores were calculated using the “ESTIMATE” R package (Yoshihara et al., 2013). The infiltration level and immune scores were compared between the high-risk group and the low-risk group.

### Immunohistochemical Analysis of Glycolysis-Related Genes in PDAC

Human protein mapping (HPA) (https://www.proteinatlas.org/) (Uhlén et al., 2015; Uhlen et al., 2017; Thul et al., 2017) of 26,000 human proteins tissue and cell distribution information. In this database, the researchers used highly specific antibodies and immunoassay techniques to examine the expression of proteins in cell lines, tumor tissues and normal samples in detail. The protein expression of 11 genes in PDAC tissues was analyzed using HPA database.

### Statistical Analysis

Most statistical analysis is done through online bioinformatics databases and tools. Other statistical analysis was performed based on R software v3.6.1. When the data is normally distributed, the mean and median of continuous variables are compared by Student's *t*-test, otherwise, use Wilcoxon inspection. The Chi-square test and Fisher test was used to compare clinical and pathological parameters and other categorical variables. Survival rates were assessed using Kaplan-Meier curves and the log-rank test, and univariate and multivariate Cox regression were used to analyze the independent parameters associated with the OS. All tests were bilateral, and *P* < 0.05 was considered statistically significant. Pearson coefficient of correlation was calculated to measure the correlation between two variables.

## Results

### Identification of Glycolysis-Related Genes in TCGA-PAAD

**Figure 1:**
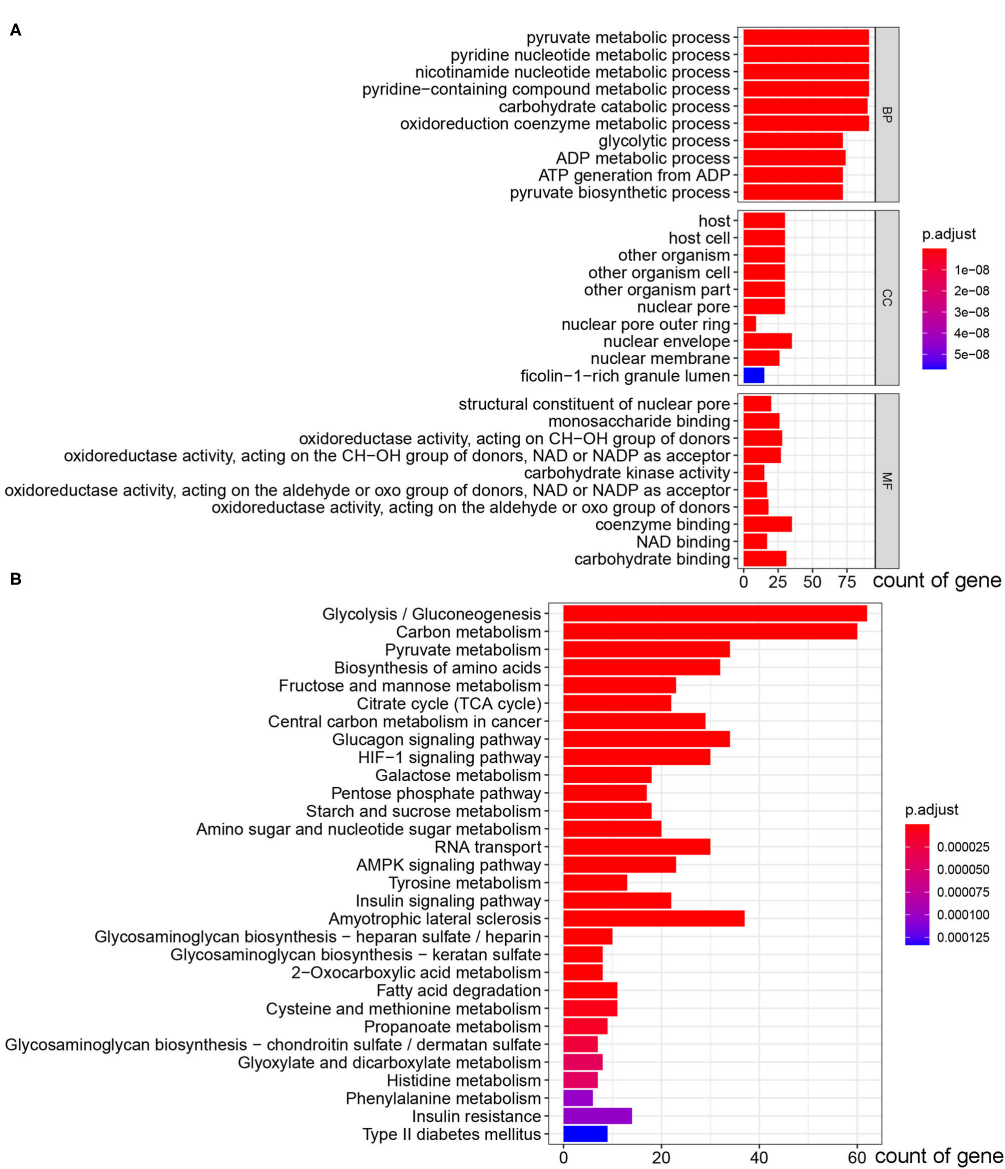
GO and KEGG pathway enrichment analysis of 319 glycolysis-related genes selected from GSEA. **(A)** The bar plot of GO pathway enrichment analysis. **(B)** The bar plot of KEGG pathway enrichment analysis (BP, biological process; CC, cell component; MF, molecular function).

A total of 13 glycolysis-related genes sets were retrieved (Table 1). These 13 gene sets contain genes involved in glycolysis, gluconeogenesis and the tricarboxylic acid cycle, metabolism of fructose 1, 6-bisphosphate and fructose 2, 6-bisphosphate, transmembrane transport of lactic acid and other biochemical reactions, which are considered to be up-regulated genes for glycolysis. After summarizing the genes in the above gene sets, 319 glycolysis-related genes were included through deduplication. To verify whether these 319 genes were involved in glycolysis, we conducted in-depth studies using GO and KEGG pathway enrichment analysis. The results showed that the genes enriched in molecular function (MF) term were related to monosaccharide binding and oxidoreductase activity, and that in biological process (BP) term were related to glycolytic metabolism, and the KEGG pathway enrichment analysis involved glycolysis/gluconeogenesis, suggesting that these genes were indeed related to glycolysis (Figures 1A,B).

**Table 1:** Thirteen glycolysis-related gene sets screened from MSigDB. Standard name: The name of genes sets in MSigDB.

**Standard name**	**Brief description**
BIOCARTA FEEDER PATHWAY	Feeder pathways for glycolysis
BIOCARTA GLYCOLYSIS PATHWAY	Glycolysis pathway
HALLMARK GLYCOLYSIS	Genes encoding proteins involved in glycolysis and gluconeogenesis
KEGG GLYCOLYSIS GLUCONEOGENESIS	Glycolysis/gluconeogenesis
MODULE 306	Glycolysis and TCA cycle
REACTOME GLYCOLYSIS	Glycolysis
REACTOME REGULATION OF GLYCOLYSIS BY FRUCTOSE 2 6 BISPHOSPHATE METABOLISM	Regulation of glycolysis by fructose 2,6-bisphosphate metabolism
WP COMPUTATIONAL MODEL OF AEROBIC GLYCOLYSIS	Computational model of aerobic glycolysis
WP GLYCOLYSIS AND GLUCONEOGENESIS	Glycolysis and gluconeogenesis
WP HIF1A AND PPARG REGULATION OF GLYCOLYSIS	HIF1A and PPARG regulation of glycolysis
GO LACTATE TRANSMEMBRANE TRANSPORT	The process in which lactate is transported across a membrane
GO LACTATE TRANSMEMBRANE TRANSPORTER ACTIVITY	Enables the transfer of lactate from one side of a membrane to the other
GO FRUCTOSE 1 6 BISPHOSPHATE METABOLIC PROCESS	The chemical reactions and pathways involving fructose 1,6-bisphosphate

### PDAC Subclasses Identification Based on Glycolysis-Related Genes

**Figure 2:**
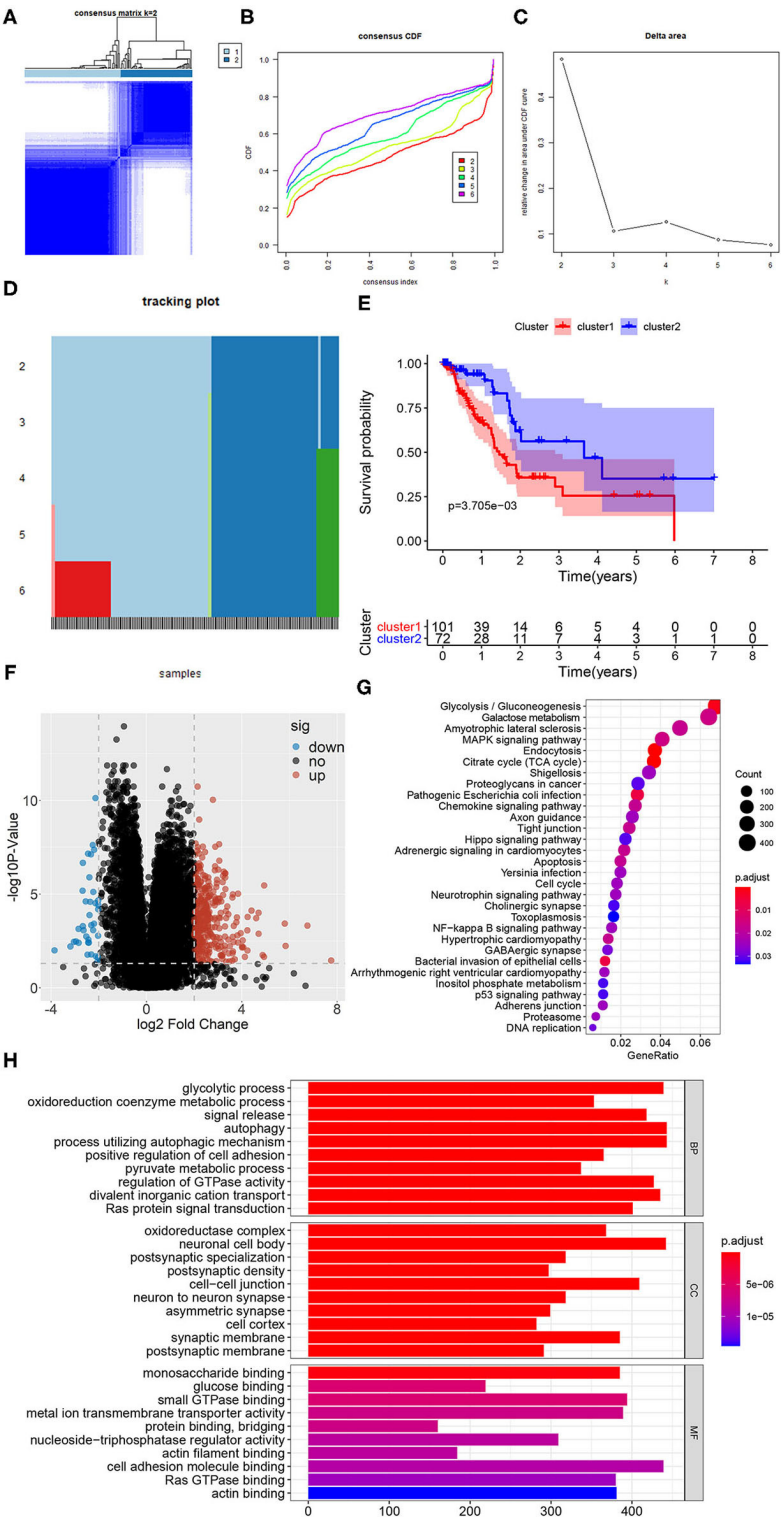
Identification of subclasses identification based on 319 glycolysis-related genes using NMF consensus clustering. **(A)** Consensus matrix heatmap for *k* = 2. **(B)** The CDF value of consensus index. **(C)** Relative change in area under CDF curve for *k* = 2–6. **(D)** The tracking plot for *k* = 2–6. **(E)** Kaplan-Meier survival analysis of PDAC patients in cluster 1 and cluster 2. **(F)** The volcano plot of the DEGs expression signature in cluster 1 and cluster 2. In the plot, “down” means down-regulated DEGs, “up” means up-regulated DEGs and “no” means the difference was not statistically significant. **(G)** The bubble plot of KEGG pathway enriched with DEGs. **(H)** The bar plot of GO pathway enriched with DEGs (BP, biological process; CC, cell component; MF, molecular function).

These 319 glycolysis-related genes were used for NMF cluster analysis, and the comprehensive correlation coefficient was used to determine the optimal *k* value as 2. TCGA-PAAD samples were then divided into two different clusters, namely cluster 1 (*n* = 101) and cluster 2 (*n* = 72). When *k* = 2, the consensus matrix heatmap had clear boundary and minimal interference between subgroups, indicating that the samples had stable clusters (Figures 2A–D). In addition, we compared the differences of survival between two clusters with different glycolytic status. The survival curve showed that patients in cluster 1 had the worse prognosis than those in cluster 2 (*P* = 0.004) (Figure 2E).

In order to better understand the glycolysis status between the samples of the two clusters, the volcano plot of DEGs of the two clusters were shown in Figure 2F, in which 343 genes were up-regulated and 37 genes were down-regulated significantly in the cluster 1 (*P* < 0.05). And the results of gene function and pathway enrichment analysis showed that these DEGs were significantly related to glycolysis and cell proliferation and migration, such as glycolytic process, oxidoreduction coenzyme metabolic process, oxidoreductase complex, glucose binding, glycolysis, citrate cycle (TCA cycle), cell cycle, autophagy and p53 signaling pathway (Figures 2G,H). In addition, we compared the infiltrating abundance of TIICs between PDAC samples in cluster 1 and cluster 2. The results showed that there were more infiltration of macrophages M0 and less infiltration of B and T cells in cluster 1 (*P* < 0.01) (Supplementary Figure 1A). Besides, our analysis also found that the samples in cluster 1 had lower immune score, stromal score, and ESTIMATE score (*P* < 0.05), while the tumor purity was higher (*P* < 0.001) (Supplementary Figures 1B–E).

### Gene Signature Identification and Score Construction

**Figure 3:**
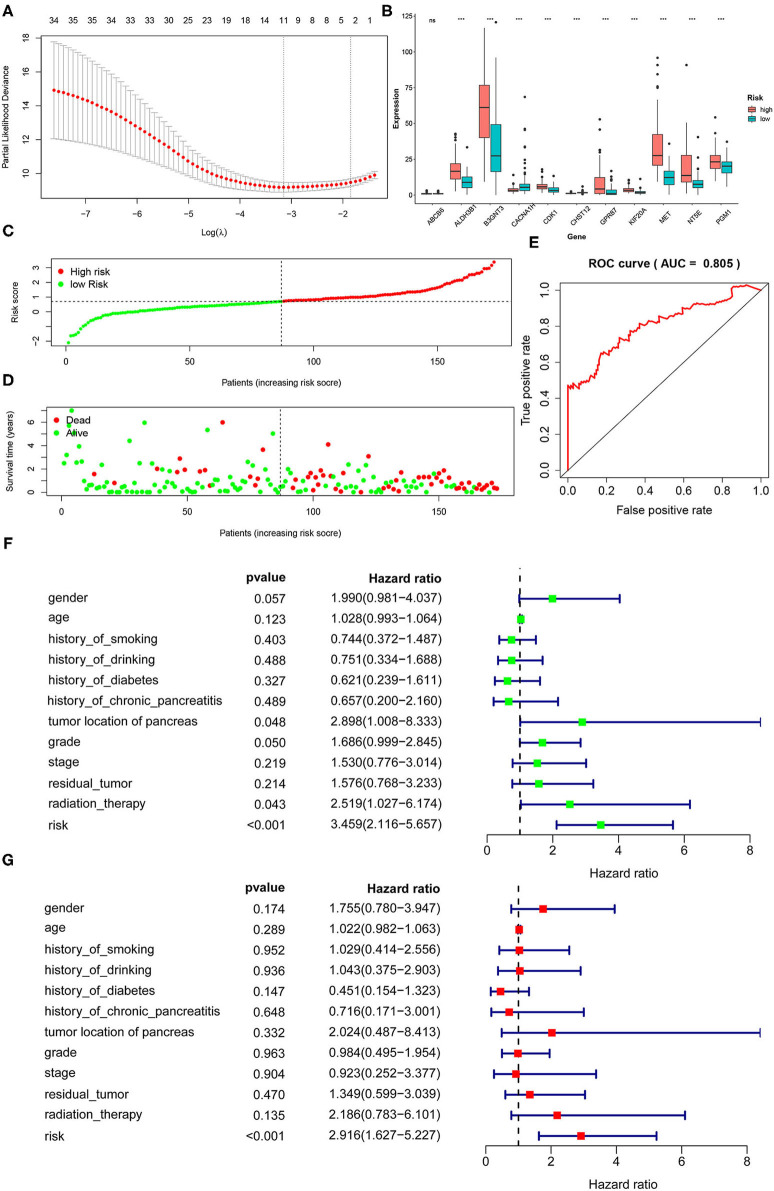
Identification of a 11-gene risk signature for PDAC patients by LASSO regression analysis. **(A)** LASSO Cox regression was used to select the most powerful parameter with cross-validation. **(B)** The heatmap of the 11 glycolysis-related gene expression signatures in the high-risk group and low-risk group. **(C)** The distribution of risk score. **(D)** The plot of survival status. **(E)** The ROC based on risk score (The risk score was divided into high-risk group and low-risk group with a cut-off value of 50%.). **(F)** Tree diagram of a univariate regression analysis. **(G)** Tree diagram of a multivariate regression analysis (ns., not significant; *P < 0.05, **P < 0.01, ***P < 0.001) (Patients with tumors located in the body and tail of the pancreas received distal pancreatectomy, and patients with tumors located in the head of the pancreas received Whipple surgery.).

The screening process for prognostic glycolysis-related genes was shown in Supplementary Figure 2. The K-M survival curves showed that among the 319 glycolysis-related genes, the expression level of 38 genes was significantly correlated with the OS of PDAC (*P* < 0.01). Among them, 30 genes were significantly upregulated in PDAC patients with poor prognosis (Supplementary Figure 3), and the high expression of 8 genes may indicate a better prognosis in patients with PDAC (Supplementary Figure 4). In TCGA-PAAD patients, 35 genes associated with total survival were identified by univariate Cox regression analysis (*P* < 0.05) (Table 2). To determine the most powerful prognostic markers, the LASSO regression analysis was used to screen 11 genes and construct risk score signature (Figure 3A) to minimize the risk of overfitting. The risk score of PAAD patients was calculated according to the expression level of gene and regression coefficient, and the results were as follows: Risk score = 0.00559311^*^ (the expression level of ALDH3B1) + 0.021443237^*^ (the expression level of PGM1) + 0.0246051^*^ (the expression level of MET) + 0.049373852^*^ (the expression level of KIF20A) + 0.036135631^*^ (the expression level of ABCB6) + 0.012568672^*^ (the expression level of NT5E) + 0.512999189^*^ (the expression level of CHST12) + 0.001510137^*^ (the expression level of GPR87) + 0.036407687^*^ (the expression level of CDK1) + 0.000281881^*^ (the expression level of B3GNT3) + 0.01548257^*^ (the expression level of CACNA1H). LASSO Cox regression fitted 11 most powerful genes into a formula related to prognosis. The PDAC patients were divided into a low-risk group (*n* = 87) and a high-risk group (*n* = 86) based on the median risk score. The expression levels of 11 prognostic markers in the high-risk group and the low-risk group were shown in a box plot (Figure 3B). As can be seen, compared with the low-risk group, ALDH3B1, PGM1, MET, KIF20A, NT5E, GPR87, CDK1, B3GNT3 were significantly up-regulated in the high-risk group (*P* < 0.001), and CHST12, CACNA1H were significantly down-regulated in the high-risk group (*P* < 0.001), while the expression level of ABAC6 was not significantly different between the two groups. Compared with the low-risk group, the high-risk group had more deaths by the time of follow-up, and the distribution of survival status and risk score was shown in Figures 3C,D. The ROC curve showed a good predictive ability of the model (AUC = 0.805, *P* < 0.001) based on the gene signature to predict the prognosis of PDAC patients (Figure 3E).

**Table 2:** Prognosis-related glycolytic genes in pancreatic cancer based on univariate Cox regression analysis.

**Gene ID**	**HR**	**95% confidence interval**	***P*-value**
ALDH3B1	1.043	1.019–1.067	0.000339
DEPDC1	1.363	1.077–1.725	0.009978
CD44	1.027	1.015–1.040	1.74E-05
NUP160	1.264	1.087–1.471	0.00236
PHKA2	0.750	0.620–0.907	0.003105
PRKACA	0.896	0.835–0.962	0.002368
HMMR	1.396	1.212–1.608	3.57E-06
PGM1	1.067	1.033–1.102	8.05E-05
B4GALT1	1.012	1.005–1.019	0.001039
AURKA	1.090	1.027–1.158	0.004705
PYGL	1.068	1.032–1.106	0.00018
PYGB	1.003	1.000–1.007	0.039495
PGK1	1.007	1.002–1.012	0.009788
MET	1.046	1.033–1.060	3.98E-12
B3GAT1	0.283	0.081–0.990	0.048251
KIF20A	1.329	1.189–1.486	5.46E-07
RARS1	1.112	1.040–1.188	0.001751
CENPA	1.227	1.078–1.395	0.001907
ABCB6	0.469	0.252–0.874	0.017151
P4HA1	1.011	1.002–1.021	0.022624
LDHA	1.011	1.007–1.016	1.38E-06
NT5E	1.040	1.026–1.053	2.75E-09
CHST12	0.305	0.174–0.537	3.73E-05
GPR87	1.049	1.026–1.073	2.64E-05
NUP54	1.198	1.022–1.403	0.025428
LHX9	1.432	1.138–1.545	0.003413
EGFR	1.047	1.012–1.083	0.007303
AK4	1.076	1.009–1.146	0.024863
CDK1	1.201	1.108–1.302	9.18E-06
B3GNT3	1.015	1.006–1.024	0.00069
COPB2	1.049	1.007–1.092	0.021589
PPIA	1.013	1.002–1.025	0.018752
CACNA1H	0.820	0.728–0.924	0.001126
RPE	1.103	1.052–1.157	4.83E-05
ERO1A	1.016	1.005–1.026	0.002958

To the diagnosis of age, gender, history of smoking, history of alcohol, history of diabetes, history of chronic pancreatitis, tumor site, histological grade, pathological stage, residual tumor and radiation therapy together with risk score into the univariate and multivariate Cox regression analysis, the results showed that the risk score based on 11 glycolysis-related genes was an independent prognostic factor in PDAC patients (*P* < 0.001, HR = 2.916, 95% CI: 1.627–5.227) (Figures 3F,G).

### Comparison of Clinicopathological Characteristic Among Risk Groups

**Figure 4:**
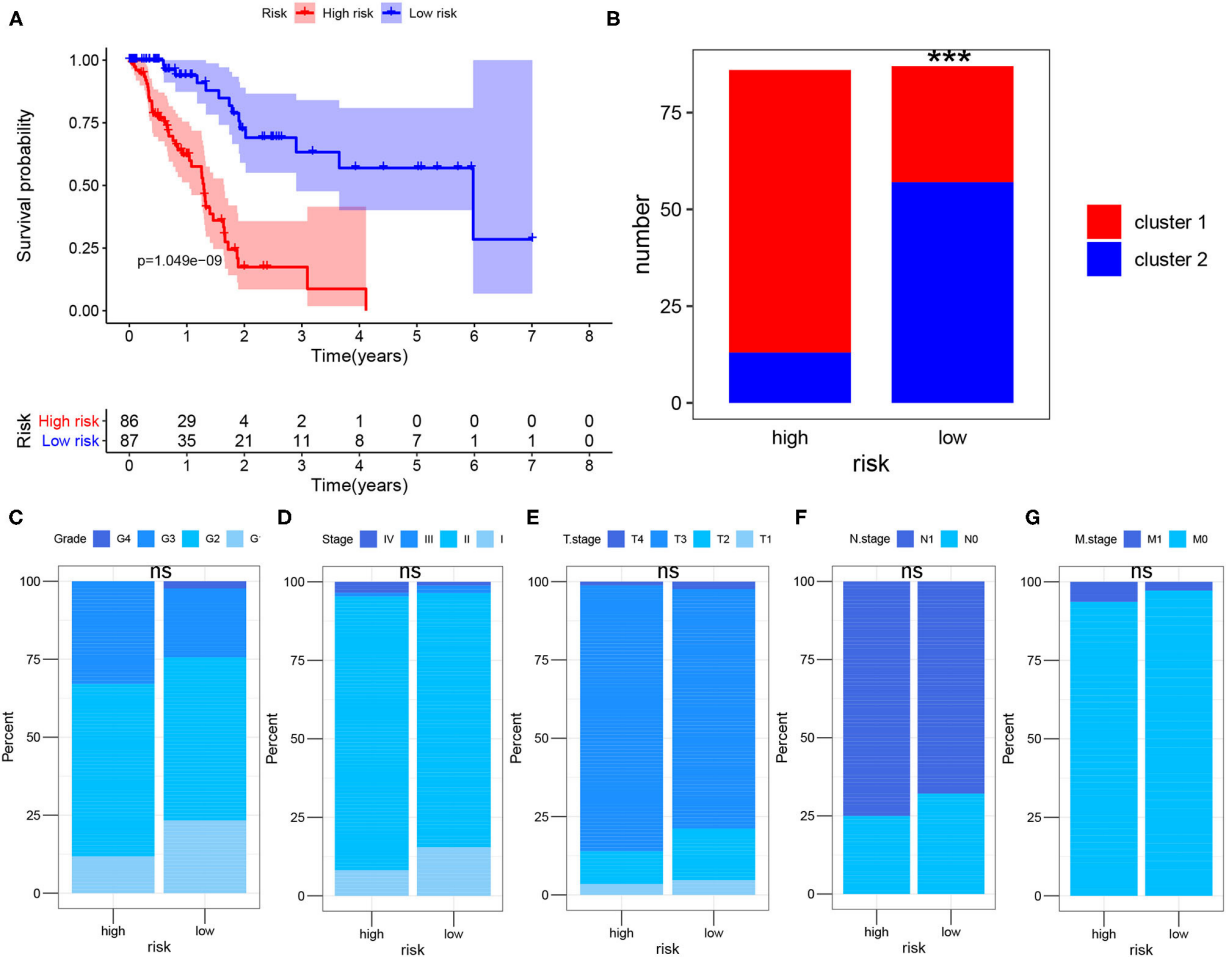
Survival analysis after risk assessment. **(A)** Kaplan-Meier survival analysis of PDAC patients in the high-risk group and low-risk group of TCGA cohort. **(B)** Sample composition of cluster 1 and cluster 2 in the high-risk group and low-risk group. **(C–G)** Comparison of tumor composition with different histological grade and pathological stage between the two groups. The Chi-square test and Fisher test were used to analyze whether the composition of clusters, T, N, M, Stage and Grade were different between the high-risk group and the low-risk group (cluster 1 vs. cluster 2, T1-2 vs. T3-4, N0 vs. N1, M0 vs. M1, Stage1-2 vs. Stage3-4, Grade1-2 vs. Grade3-4; ns., not significant; ***P < 0.001).

To further evaluate the impact of risk score on prognosis of PDAC patient, K-M analysis showed that the prognosis of high-risk group was significantly worse than that of low-risk group (*P* < 0.001) (Figure 4A). In addition, we found that the high-risk group included more patients in cluster 1 (cluster 1: cluster 2 = 72:14), and the low-risk group mainly included patients in cluster 2 (cluster 1: cluster 2 = 29:58), the difference was statistically significant (*P* < 0.001) (Figure 4B), indicating that patients in the high-risk group had a more active glycolysis status. Besides, we compared the differences in the pathological stage and histological grade of the cancers between the high-risk group and the low-risk group in detail. We found that although there were no statistically significant differences in T, N, M, Stage and Grade composition between the high-risk group and the low-risk group (*P* > 0.05), the high-risk group tended to include more PDAC samples with late stage and high grade (Figures 4C–G).

### External Validation of the Gene Signature

**Figure 5:**
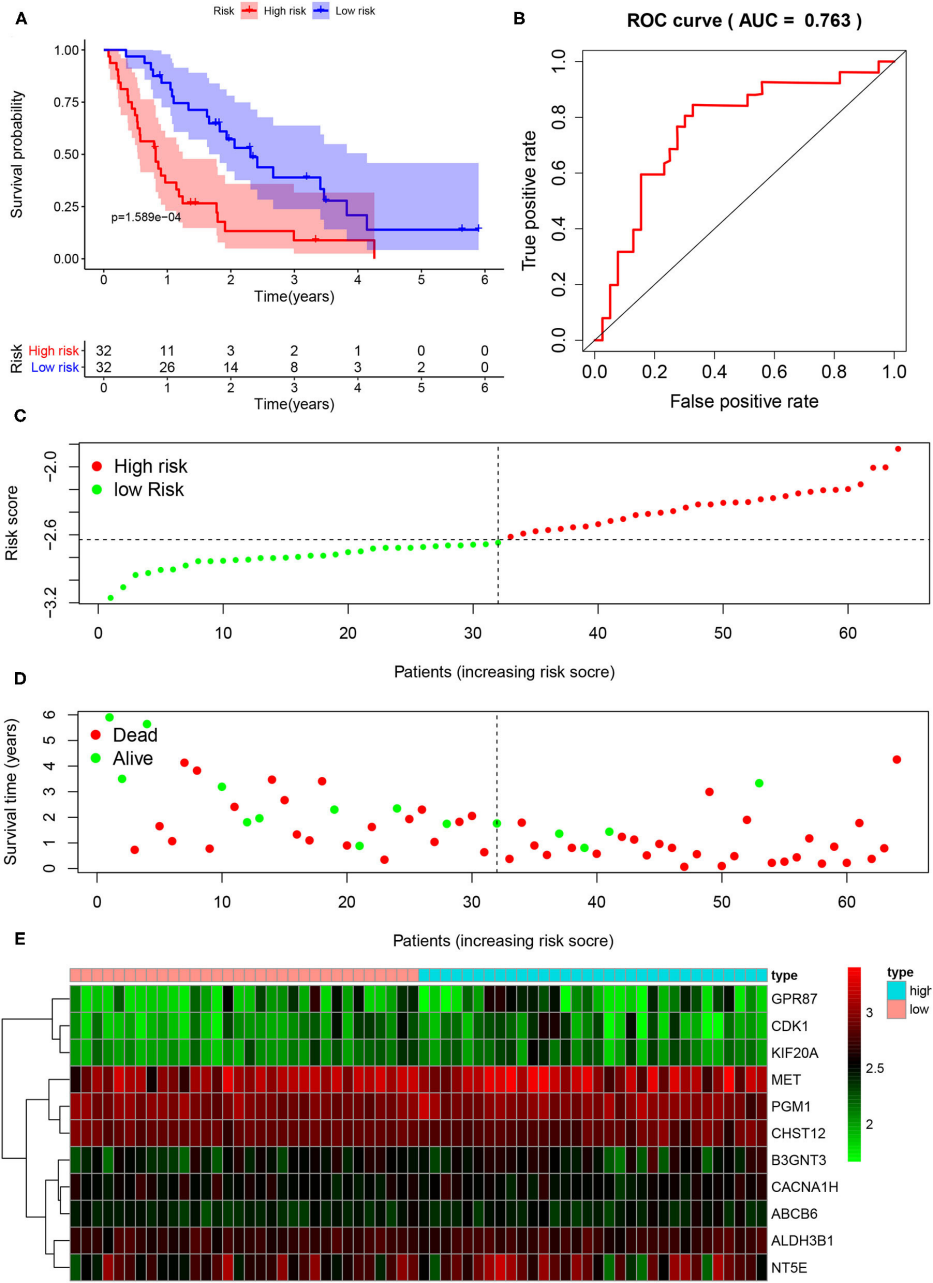
External validation of the risk prediction model using the GEO dataset. **(A)** Kaplan-Meier survival analysis of PDAC patients in the high-risk group and low-risk group of GEO dataset. **(B)** The ROC based on risk score. **(C)** The distribution of risk score. **(D)** The plot of survival status. **(E)** The heatmap of the 11 glycolysis-related gene expression signatures in the high-risk group and low-risk group.

To further verify the usefulness of the risk score model based on 11 glycolysis-related gene signatures, we downloaded the GSE62452 (*n* = 130) data set from GEO, in which 69 samples were PDAC samples. After removing samples whose gene expression was 0 and samples without survival data, a total of 64 samples were included in the study. According to the above formula, the risk score of the GEO dataset sample was calculated and the patients were divided into the low-risk group (*n* = 32) and the high-risk group (*n* = 32). K-M analysis showed that the high-risk group had a significantly worse prognosis than the low-risk group (Figure 5A). Compared with the low-risk group, the high-risk group had more deaths by the time of follow-up. The distribution of survival status and risk score of the patients was shown in Figures 5C,D. Predicting the prognosis of PDAC patients based on this gene signature, the ROC curve showed a good predictive ability of the model (AUC = 0.763, *P* < 0.001) (Figure 5B). The expression levels of 11 prognostic markers in the high-risk group and the low-risk group were shown by heatmap (Figure 5E).

### Function and Mechanism Inference

**Figure 6:**
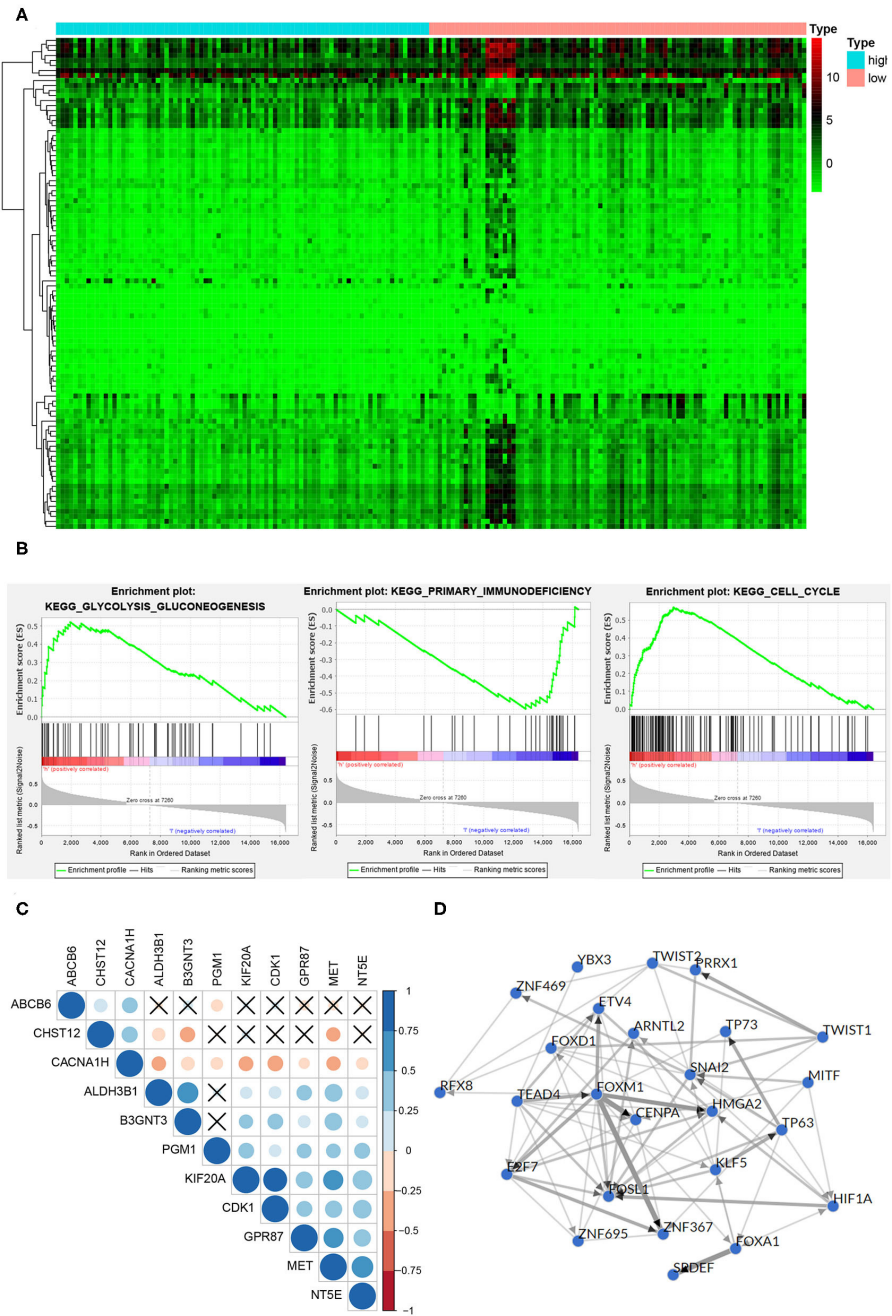
Function and mechanism inference of DEGs in the high-risk group and low-risk group. **(A)** The heatmap of the DEGs expression signature in the high-risk group and low-risk group. **(B)** The DEGs were enriched in glycolysis gluconeogenesis, primary immunodeficiency and cell cycle pathway. **(C)** TF networks of 11 glycolysis-related genes. Red represented a positive correlation, blue represented a negative correlation, and ×represented no significant correlation. **(D)** The correlation plot of 11 glycolysis-related genes. The thickness of the line indicated the correlation.

The DEGs between the high-risk group and the low-risk group were shown in the heatmap (Figure 6A), in which 440 genes were significantly up-regulated and 25 genes were significantly down-regulated in the high-risk group (*P* < 0.05). It is worth noting that GSEA analysis results showed the DEGs were enriched in glycolysis gluconeogenesis, primary immunodeficiency and cell cycle pathway (Figure 6B), which meant that there were differences in glycolysis levels, immune status, and tumor cell proliferation in the high and low risk groups. The correlation plot showed that there were strong positive correlations between MET and other oncogenic glycolysis genes, such as KIF20A and MET (*r* = 0.52961), NT5E and MET (*r* = 0.634197), GPR87 and MET (*r* = 0.522604), etc. (*P* < 0.001), while there were negative correlations between MET and ABCB6, CHST12 and CACNA1H (Figure 6C). Figure 6D depicted a network of top-25 TF with strong regulatory relationships among 11 genes, including HIF-1A.

### Protein Expression Analysis of Glycolysis-Related Genes

**Figure 7:**
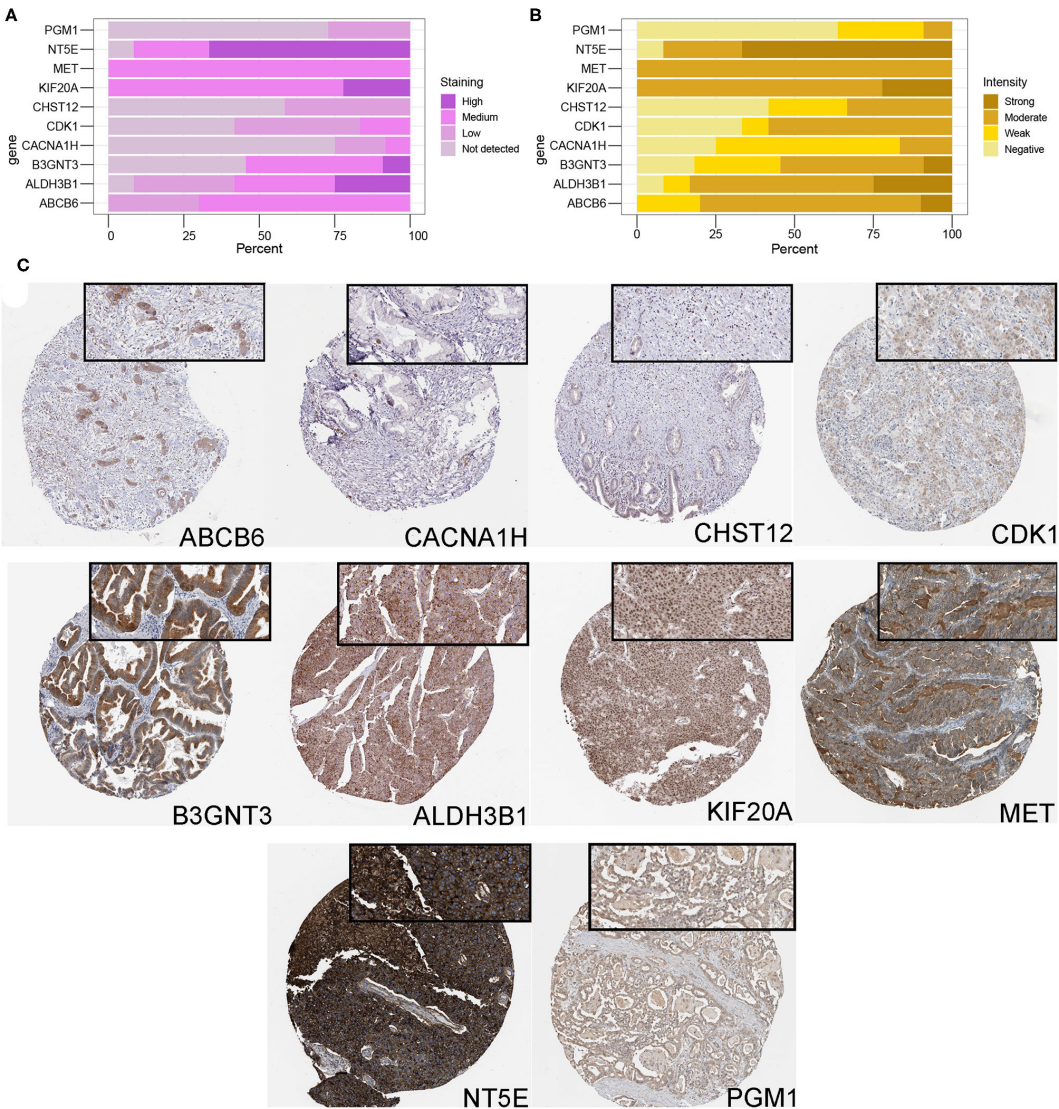
Protein expression analysis of glycolysis-related genes. **(A,B)** The protein expression score of staining and intensity of 10 proteins in all PDAC samples. Protein expression score is based on immunohistochemical data manually scored with regard to staining intensity (negative, weak, moderate or strong) and fraction of stained cells (<25%, 25–75% or >75%). Percentage represented the percentage of each protein expression score and intensity level in all samples. **(C)** Immunohistochemical staining of ABCB6, CHST12, CACNA1H, ALDH3B1, PGM1, MET, KIF20A, NT5E, CDK1, and B3GNT3 proteins in PDAC samples.

Finally, in order to study the expression level of glycolysis gene in PDAC samples at the protein level, immunohistochemical data of 10 proteins were retrieved from the HPA database, and immunohistochemical information of GPR87 was lacking. Figures 7A,B showed the staining and intensity of 10 proteins in all PDAC samples. The results showed that ABCB6, CHST12, and CACNA1H were weakly stained in PDAC samples, while ALDH3B1, PGM1, MET, KIF20A, NT5E, CDK1, and B3GNT3 were strongly stained and the protein level was higher in PDAC samples (Figure 7C), consistent with that at mRNA level.

### Analysis of Immune Cell Infiltration in PDAC

The box plot showed the infiltrating abundance of TIICs with significant differences (*P* < 0.05) in PDAC samples between the high-risk and low-risk groups. It can be seen that, compared with the low-risk group, the infiltration abundance of macrophages in the high-risk samples was higher, and that of CD8+T cells was lower, which was the same as in cluster 1 (Supplementary Figure 5A). In addition, our analysis also found that the high-risk group had lower immune score, stromal score, and ESTIMATE score (*P* < 0.05), while the tumor purity was higher (*P* < 0.05) (Supplementary Figures 5B–E).

## Discussion

PDAC is a common cause of cancer-related death and has a low survival rate. It is estimated that there will be 57,600 new cases and 47,050 deaths in the United States in 2020 (Siegel et al., 2020). Many methods can be used in the clinical treatment of PDAC, such as surgery, chemotherapy, radiotherapy, immunotherapy, targeted molecular therapy, etc. The individual characteristics of PDAC patients are of great significance for precision therapy, among which the genetic characteristic of tumor is a very important aspect. There have been many studies on gene signatures based on TCGA and GEO database to predict the prognosis of PDAC patients, including immune-related genes and mucin genes (Wei et al., 2019; Wu et al., 2019, 2020; Jonckheere et al., 2020). Pilar Espiau-Romera's review summarized the correspondence between the molecular subtypes and metabolic subtypes of PDAC, and the metabolic reprogramming involved in PDAC included lipid metabolism, glycolysis, and amino acid consumption. The survival analysis showed that the subtype classification of PDAC based on metabolism was more significant for clinical diagnosis and treatment, because it can not only indicate the prognosis of patients, but also reflect the invasion, drug resistance and other biological characteristics of PDAC (Espiau-Romera et al., 2020). In this study, we used the TCGA-PAAD cohort to analyze the effect of glycolysis status on the OS of PDAC patients, suggesting the potential value of glycolysis-related gene signature as prognostic markers.

First, we extracted glycolysis-related genes by searching the gene sets from GSEA, and then according to these 319 gene expression levels, the PDAC samples were divided into 2 clusters by NMF cluster analysis. It was found that cluster 1 contained more advanced samples and the prognosis of the patients was worse. Further, 38 prognostic genes were selected by K-M survival analysis and a risk scoring model based on LASSO regression analysis. ALDH3B1, PGM1, MET, KIF20A, NT5E, GPR87, CDK1, B3GNT3 were considered to play a role in promoting cancer, while CHST12, CACNA1H, and ABAC611 were thought to inhibit tumor progression, and similar expression differences were found in the protein level. Correlation analysis suggested that MET had a strong positive correlation with other glycolysis-related genes. Current research has shown that MET can establish connections between extracellular matrix and cytoplasm by binding to its ligand, hepatocyte growth factor (HGF). In cancer cells, abnormal HGF/C-MET axis promoted tumor progression by inducing PI3K/AKT, Ras/MAPK and other signaling pathways (Hervieu and Kermorgant, 2018; Zhang et al., 2018). Yan et al. demonstrated that HGF/C-met enhances the stem-like potential and glycolysis of PDAC cells by activating YAP/HIF-1 (Yan et al., 2018). ALDH3B1 was a member of the ALDH family and was generally considered to be metabolically active, with unique specificity for various aldehyde substrates (Kitamura et al., 2013). Expression pattern and clinical significance studies have found that ALDH3B1 was significantly highly expressed in lung cancer (Marchitti et al., 2010), and can be an independent prognostic factor for lung cancer (Sun et al., 2020). PGM1 was an enzyme in the glycogen degradation pathway responsible for converting glucose 1 phosphate into glucose 6 phosphate. Marion et al. found that mutations in PGM1 can block its enzyme activity and prevent cancer-related fibroblast stimulation of glycogen mobilization (Curtis et al., 2019). Jung et al. found that KIF20A was up-regulated in a lactate-dependent manner to promote metastasis in the presence of excess lactic acid resulting from enhanced aerobic glycolysis in cancer (Jung et al., 2019). Yu et al. have incorporated NT5E into the glycolytic-based seven-gene signature of gastric cancer, which was closely related to the prognosis of gastric cancer patients and tumor immune infiltration (Yu et al., 2020). GPR87, CDK1, and B3GNT3 were significantly overexpressed in PDAC cells and clinical tissues, indicating poor prognosis (Wang et al., 2017; Mishra et al., 2019; Piao et al., 2019). There have also been previous studies on glycolysis-related genes in PDAC. For example, Tian et al. screened 13 glycolysis genes as independent prognostic factors in PDAC patients through multivariate Cox regression analysis and survival analysis, and constructed forward stepwise Cox regression model to calculate the risk score, which was 0.700 × Met + 0.683 × B3GNT3 + 0.662 × SPAG4. Next, risk score, age, sex, tumor stage, radiotherapy, and residual tumor were included to establish a nomogram based on multivariate Cox regression analysis to predict the prognosis of PDAC. Our analysis and calculation results showed that a total of 11 glycolysis-related genes were included in the calculation of risk score, and compared with the three-gene model proposed by Tian et al., the prediction performance was better (AUC: 0.805 vs. 0.764). Moreover, we considered more factors related to PDAC pathogenesis and prognosis, such as, history of smoking, history of alcohol, history of diabetes, history of chronic pancreatitis, and tumor site (Tian et al., 2020).

After grouping according to the risk score, the high-risk group included more PDAC samples of late stage and high grade, and patients had a worse prognosis. External data reached the same conclusion, further demonstrating the specificity and accuracy of this genetic feature in differentiating PDAC with different prognoses. Epidemiological studies have found that history of smoking and drinking can promote the occurrence and progression of PDAC (Ezzati et al., 2005; Hidalgo, 2010; Parkin et al., 2011; Bosetti et al., 2012). The incidence of PDAC was also found to differ by sex, possibly due to smoking (Ilic and Ilic, 2016). In addition, chronic pancreatitis and diabetes were also found to be risk factors for PDAC (Wolpin et al., 2013; Andersen et al., 2017; Kirkegård et al., 2017). In addition, traditional AJCC TNM staging is currently recognized as the most effective prognostic tool for PDAC (Kamarajah et al., 2017). The tumor site of PDAC also had a significant difference in the prognosis of patients. Tumors located in the body or tail of pancreas often predicted a poorer prognosis, which may be caused by the more hidden onset, larger tumor size, higher risk of metastasis and difficulty of resection (van Erning et al., 2018; Tomasello et al., 2019). The present univariate and multivariate Cox regression analysis showed that the clinical pathological characteristics, such as age, gender, smoking history, drinking history, diabetes history, history of chronic pancreatitis, tumor location, histological Grade, pathological stage, residual tumor and radiation therapy had no significant effect on OS in PDAC patients, which may be due to insufficient sample size. Even so, risk score was found to be an independent prognostic factor in patients with PDAC.

In addition, the high-risk group included more cluster 1 samples, suggesting that the high-risk group had a higher level of glycolysis, leading to a poorer prognosis. GSEA analysis showed that DEGs higher expressed in high-risk group were mainly concentrated in glycolysis gluconeogenesis, primary immunodeficiency and cell cycle pathway, suggesting that PDAC in high-risk group had higher level of glycolysis, cell proliferation, and immunosuppression, which is consistent with the previous theory (Yang et al., 2020). With the discovery of abnormal glucose metabolism in cancer cells, more and more drugs targeting the glycolysis process are being developed and used in clinical trials. There were drugs targeting c-MET in clinical trials, such as onartuzumab, crizotinib, and tivantinib (Zhang et al., 2018). The usage of PGM1 inhibitors may be a therapeutic strategy to reduce the spread of metastatic abdominal cancers, such as PDAC (Curtis et al., 2019).

Currently, immunotherapy has been effective in a variety of cancers (Brahmer et al., 2012; Borghaei et al., 2015; Motzer et al., 2015; Robert et al., 2015; Sharma et al., 2016), but has not yet been converted to PDAC (Royal et al., 2010; Le et al., 2019). Most evidence indicated that there were dense stromal cells in the microenvironment of PDAC, and the immune cells were mainly myeloid suppressor cells and tumor-associated macrophages (TAM), forming an immunosuppressive environment devoid of nutrients (Dougan, 2017). Due to the abundant infiltration of bone marrow cells and the relative lack of T cells in PDAC (Liu et al., 2016), PDAC has poor response to immunocheckpoint treatment and poor immunotherapy effect. Raghu et al. found a survival advantage in PDAC patients with T cell infiltration in the tumor by immunohistochemical staining and multispectral imaging (Carstens et al., 2017). By analyzing the infiltration of TIICs in PDAC samples, we found that, compared with the low-risk group, the infiltration level of TIICs in the high-risk group was generally lower, M0 and M1 macrophages infiltration abundance was higher, and CD8+T lymphocytes was with low abundance, which may explain the poor prognosis of high-risk group from the aspects of the immune mechanism. Moreover, the above characteristics of TIICs infiltration also existed in cluster 1 patients, indicating that the high-risk group had a strong similarity to cluster 1. Despite the difficulties of immunotherapy for PDAC, current research offered glimmers of hope, such as depletion or reprogramming of myeloid cells to reduce immunosuppression and fibrosis, and recruitment and enhancement of T cell response (Dougan, 2017).

Our study focused on bioinformatics to predict the diagnostic, therapeutic and prognostic value of glycolysis-related genes in PDAC, and explored the potential mechanisms by which glycolytic genes regulated of tumor cell invasion and migration. In addition, we analyzed the level of TIICs in PDAC and its relationship with prognosis. Our results indicated that the high level of glycolysis may suggest a poor prognosis in PDAC patients, which can be concluded from the comparison of signaling pathways and immune infiltration. However, we had to say that this study was a retrospective study with some limitations. Due to the low incidence of PDAC, the sample size collected by each study or center was small, which was a deficiency of this study. Therefore, we call for a larger sample size prospective study to verify the clinical application of glycolysis-related genes in personalized management of PDAC patients. We performed GO and KEGG analyses on these 319 genes, and the results showed that they were indeed enriched in glycolysis, however these genes may also be involved in regulating other processes. But complex biological processes in the human body resulted that nearly every gene could participate in multiple signaling pathways and perform multiple functions. Although we haven't found a suitable method to solve this problem now, but we will try to develop a more accurate method to screen genes for further analysis and research. In addition, this study inferred from the perspective of bioinformatics that there was a lack of experimental verification, such as the absence of resolution of glycolysis gene expression in PDAC cells, and the absence of functional studies to block or interact with glycolysis gene. In this regard, Li et al. also conducted a similar analysis and found that glycolysis level was associated with the prognosis of PDAC patients. It is worth learning that Li et al. verified that STAT3 signaling pathway was the key pathway regulating glycolysis in PDAC and there was a positive feedback between glycolysis level and STAT3 signaling activity at the cellular level (Li et al., 2021). As a continuation of future research, we will supplement it in future study.

In present study, we used the TCGA-PAAD RNA-seq data and clinical data, constructed risk prediction model based on glycolysis gene. The risk score based on this model was an independent prognostic factor for PDAC and can potentially predict the prognosis of patients. The risk prediction model was useful for verifying PDAC patients with poorer prognoses and might offer a new view for the research of individual treatment. In addition, the included glycolysis genes were significantly correlated with the invasion, cell division and cell adhesion and abundance of TIICs, and corresponding targeted drugs have been emerging.

## Data Availability Statement

The original contributions presented in the study are included in the article/Supplementary Material, further inquiries can be directed to the corresponding author/s.

## Author Contributions

WS and JZ contributed to the conception of the study. PG, XH, and YY contributed significantly to analysis and manuscript preparation. SH and YZ performed the data analyses and wrote the manuscript. LW helped perform the analysis with constructive discussions. All authors contributed to the article and approved the submitted version.

## Conflict of Interest

The authors declare that the research was conducted in the absence of any commercial or financial relationships that could be construed as a potential conflict of interest.

## Supplementary Material

Extended data TCGA-PAAD dataset: https://doi.org/10.6084/m9.figshare.13347536; GSE62452 dataset: https://doi.org/10.6084/m9.figshare.13347701; Code: https://github.com/songwenjing527/Glycolytic-genes-in-PDAC.git.
