# Supplementary material for: Glycolysis-Related Gene Expression Profiling Screen for Prognostic Risk Signature of Pancreatic Ductal Adenocarcinoma
Source: Front Genet. 2021 Jun 23;12:639246. doi: 10.3389/fgene.2021.639246 (PMC8261051; doi:10.3389/fgene.2021.639246)
Supplement: Extended data — TCGA-PAAD dataset: https://doi.org/10.6084/m9.figshare.13347536; GSE62452 dataset: https://doi.org/10.6084/m9.figshare.13347701; Code: https://github.com/songwenjing527/Glycolytic-genes-in-PDAC.git. [file Data_Sheet_1.docx]

Supplementary Material

# Supplementary Figures


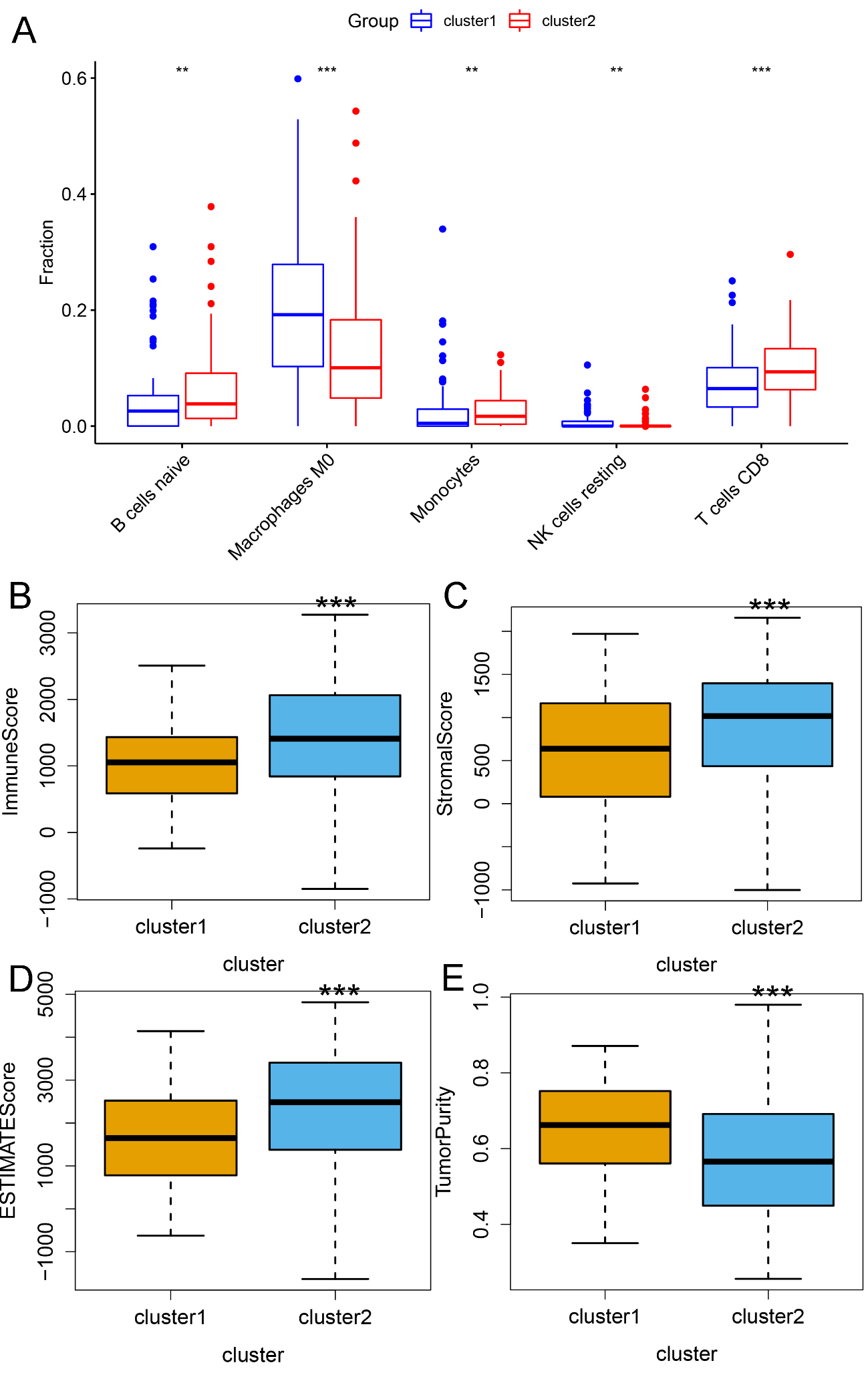


**Supplemental Figure. 1** Analysis of immune cell infiltration in cluster1 and cluster2. (A) Infiltrating immune cells with significantly different abundance in cluster1 and cluster2. (B-E) The boxplot of immune score, stromal score, ESTIMATE score, and tumor purity in cluster1 and cluster2. (* P < 0.05, ** P < 0.01, *** P < 0.001.) https://figshare.com/s/bb87388e7c92a8e8c559


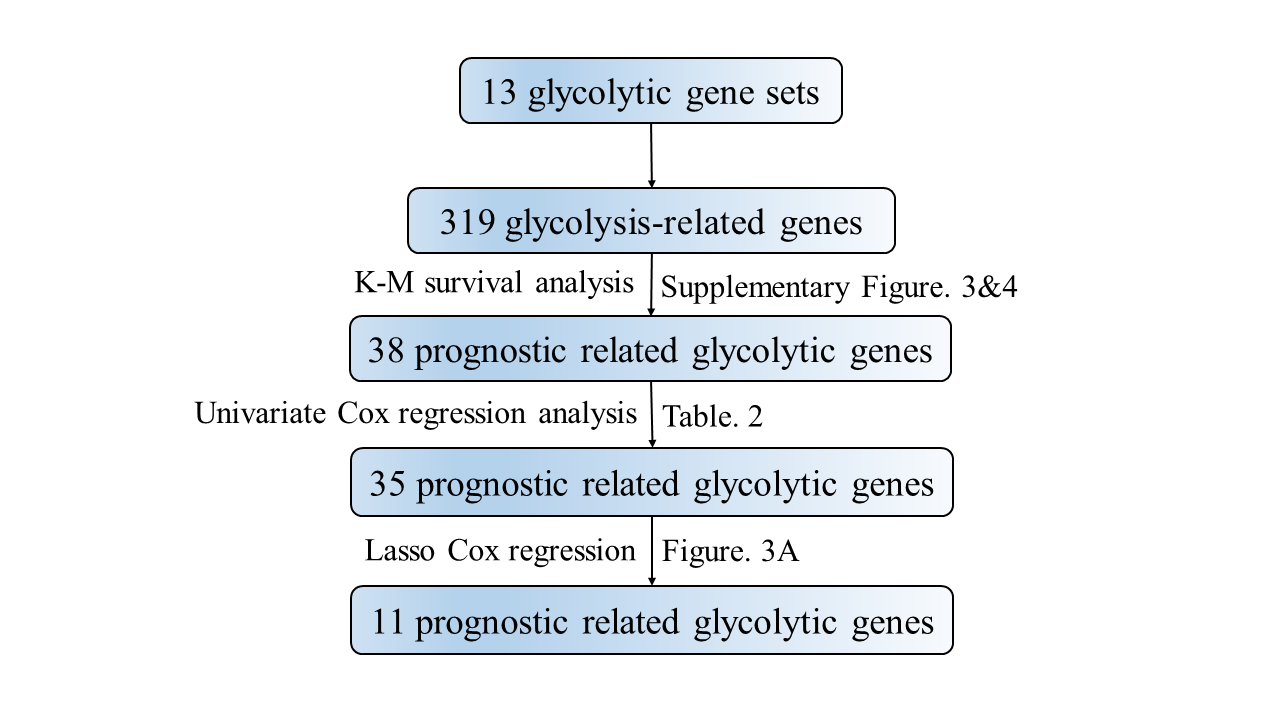
**Supplemental Figure. 2** The screening process for prognostic glycolytic genes. https://figshare.com/s/1bb6aba9a46c8e4c7692


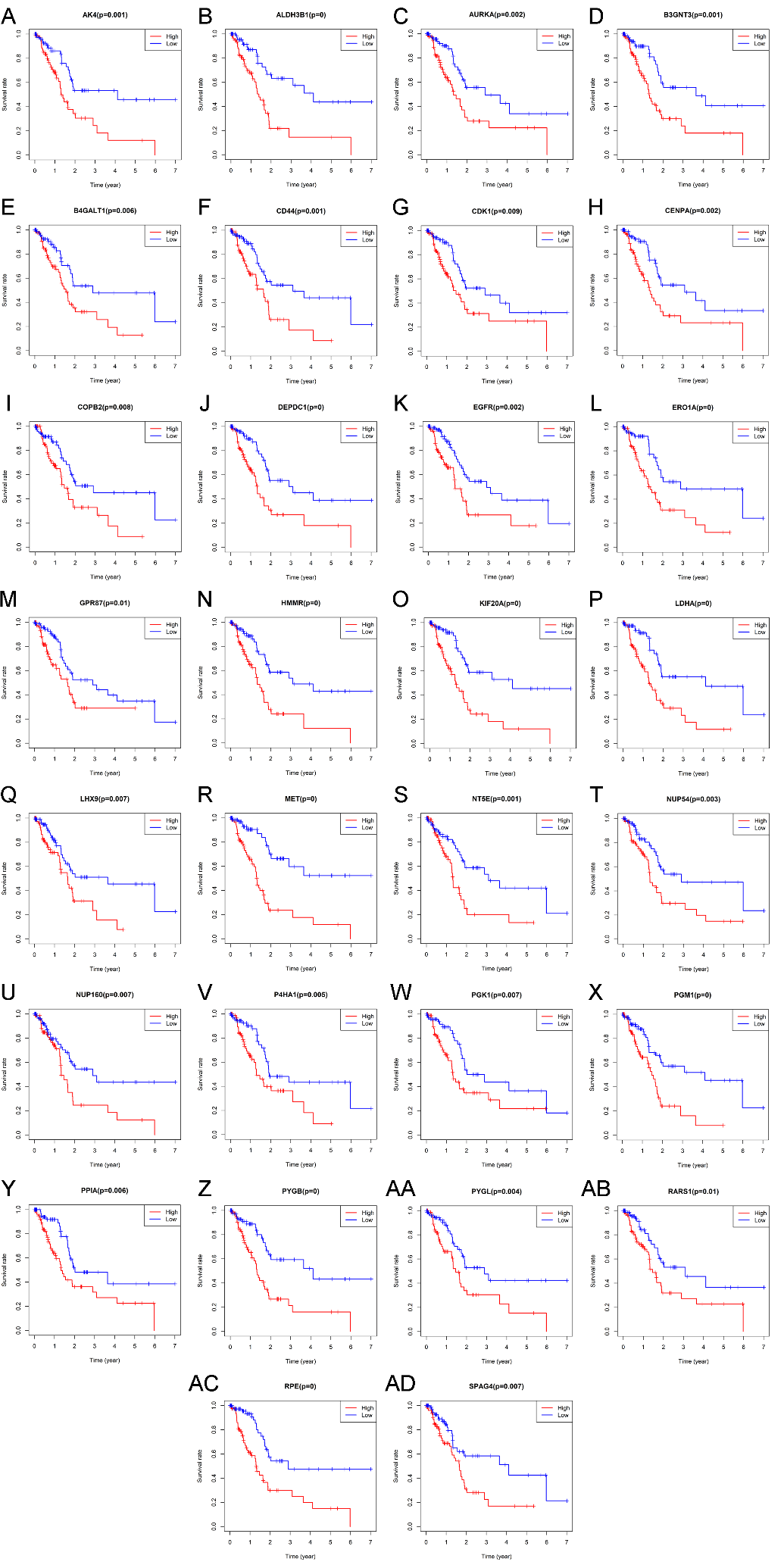


**­-Supplemental Figure. 3** The survival analysis of 30 oncogenic glycolytic genes in PDAC. (A-AD) The Kaplan-Meier OS survival curves of MET, KIF20A, ALDH3B1, RPE, LDHA, HMMR, PYGB, DEPDC1, PGM1, ERO1A, CD44, AK4, NT5E, EGFR, CENPA, AURKA, NUP54, PYGL, P4HA1, B4GALT1, PPIA, LHX9, SPAG4, PGK1, B3GAT1, NUP160, COPB2, CDK1, RARS1, GPR87 in PDAC. https://figshare.com/s/4db36c6ccab442c60b41


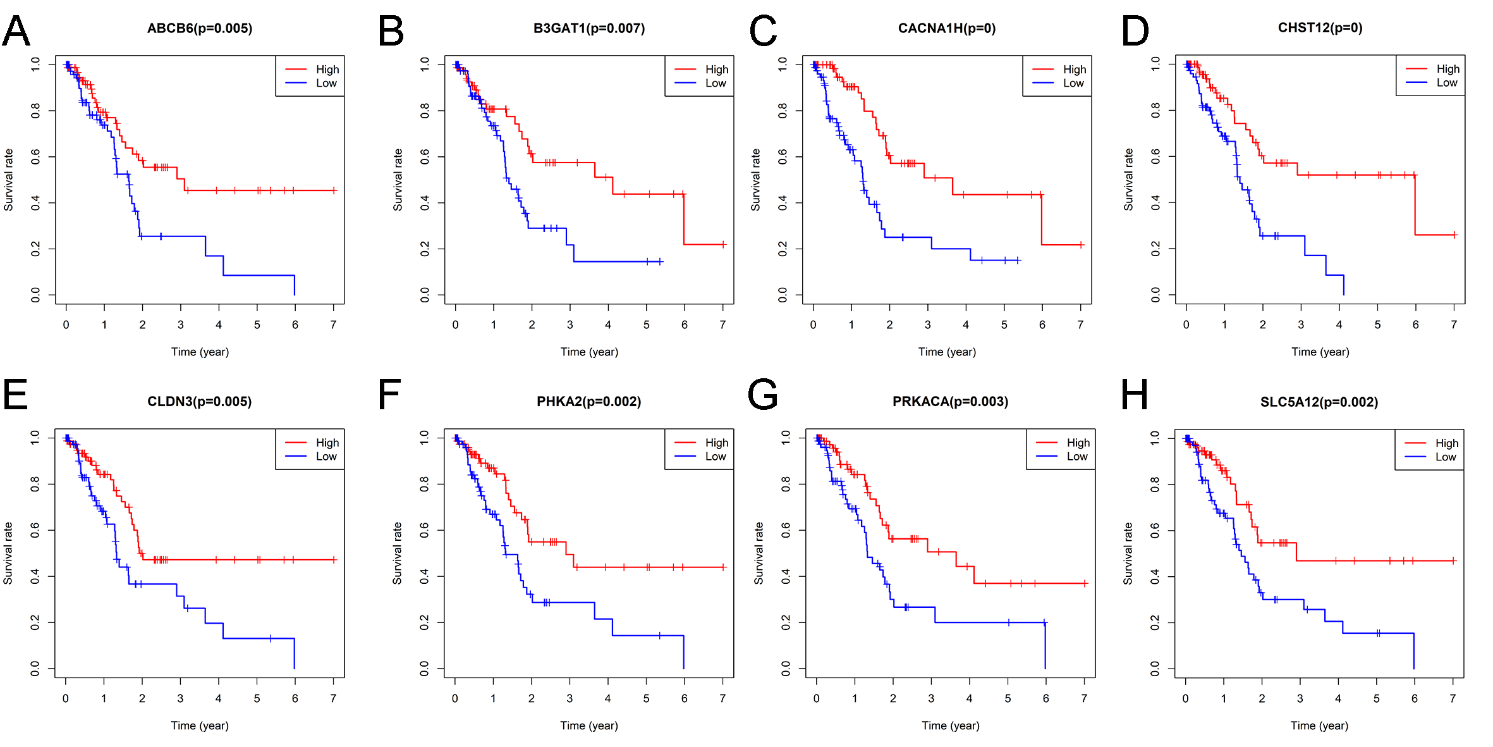
**Supplemental Figure. 4** The survival analysis of 30 cancer suppressor glycolytic genes in PDAC. (A-H) The Kaplan-Meier OS survival curves of ABCB6, B3GNT3, CACNA1H, CHST12, CLDN3, PHKA2, PRKACA, SLC5A12 in PDAC. https://figshare.com/s/afa1bb2948f5555e1a35


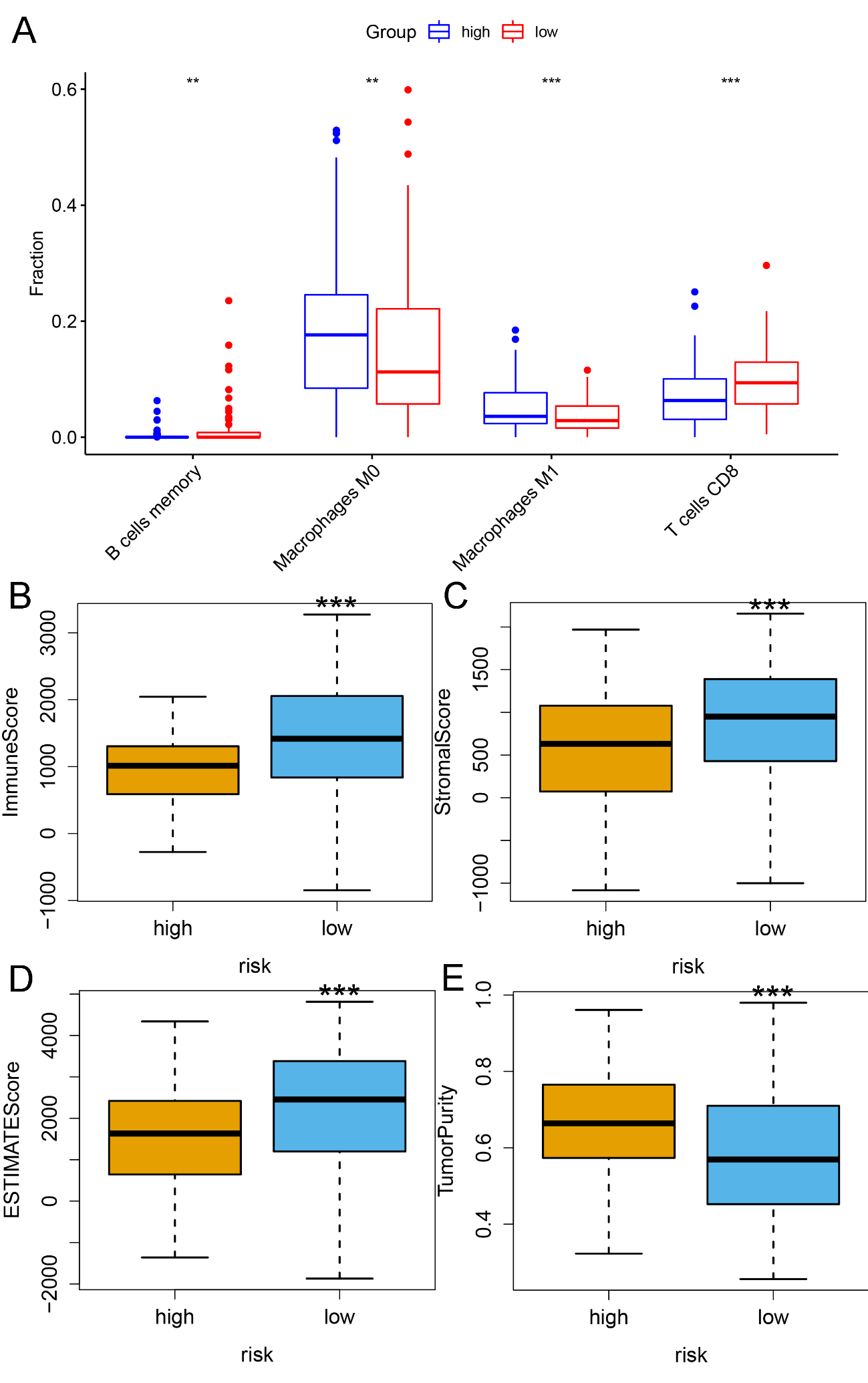


**Supplemental Figure. 5** Analysis of immune cell infiltration in high-risk and low-risk groups. (A) Infiltrating immune cells with significantly different abundance in high-risk and low-risk groups. (B-E) The boxplot of immune score, stromal score, ESTIMATE score, and tumor purity in high-risk and low-risk groups. (* P < 0.05, ** P < 0.01, *** P < 0.001.) https://figshare.com/s/0dd46f3766fa321ae414
